# Global child and adolescent mental health: challenges and advances

**DOI:** 10.1080/17571472.2018.1484332

**Published:** 2018-07-16

**Authors:** Lauren Bruha, Valentini Spyridou, Georgia Forth, Dennis Ougrin

**Affiliations:** a Department of Child and Adolescent Psychiatry, Institute of Psychiatry, Psychology and Neuroscience, King’s College London, De Crespigny Park, UK

Global mental health has never been more topical. The true global burden of mental illness has only recently been understood. Mental health disorders account for 32.4% of years lived with disability (YLDs) and 13.0% of disability-adjusted life-years (DALYs), making these disorders by far the largest contributor to the global burden of disease in terms of YLDs, and on a par with cardiovascular diseases in terms of DALYs [1].

Mental health disorders are very common. The prevalence of mental health disorders in children and adolescents is almost 15% globally [2]. With the recognition that 50% of mental health disorders begin by the age of 14 and 75% by the age of 24 [3], child and adolescent mental health has become a global priority.

The collection of the articles on global child and adolescent mental health in London Journal of Primary Care is both timely and illuminating (Yuan Dahlan, Sakano). It is based on the materials of a Global Child and Adolescent Mental Health conference at King’s College London which was convened in June 2018 to celebrate the 30th anniversary of the MSc in Child and Adolescent Mental Health, the first postgraduate course of its kind in the world.

The course currently has over 300 alumni from nearly 60 different countries. The three central topics discussed in these articles are service development, globally relevant innovative research and the need for coordinated policy to tackle stigma and achieve parity of esteem between mental and physical illness. Although the papers in this issue come from very disparate countries, most of the problems highlighted are universal.

The problem of stigma seems to be of utmost importance in Japan, Malaysia and China as well as in the UK. Whilst the issue of institutionalization and hospital-based treatment seems to be significantly greater in Japan (Sakano), it certainly has not gone away in the West and remains a focus of intense research [4]. Schools in China are ill-equipped to teach students with special needs (e.g. individuals with Autism Spectrum Disorder or a learning problem) due to the lack of training and/or personal experience with such students (Yue). And similar problems are faced by thousands of British parents unable to access good quality education for their children.

The articles in this collection also highlight important breakthroughs and achievements in child and adolescent mental health. With the prevalence of mental health disorders increasing in young people, countries like Malaysia have created and implemented the Mental Health Service Policy (Dahlan), in line with the WHO’s Comprehensive Mental Health Action Plan 2013–2020, the United States’ the 21st Century Cures Act, which embodies the Helping Families in Mental Health Crisis Act and the UK’s own Green Paper on transforming children and young people’s mental health provision. For each of these policies, the aim is to provide proper care, education and protection to all individuals with mental health difficulties. This includes population groups that are less likely to receive evidence-based treatments, such as Looked After Children. These global policy advances are relatively new and they have a real potential to prioritise child and adolescent mental health around the world.

So much more, however, could be done. Looking towards the future, a worldwide goal could be to create guidelines like the National Institute for Health and Care Excellence (NICE). Developing, implementing and maintaining these guidelines will increase consistency among mental health services. Each guideline should be country specific but should contain best-evidence treatments for each disorder. We should learn from the young people about harnessing technology to improve mental health. We could streamline our modest resources by providing online assessments and by developing internet-based therapies and supervision [5,6]. We are only at the start of a technological revolution in child and adolescent mental health and learning from global technological advances such as the use of Virtual Reality in the treatment of ASD (Yuan) is vital. And who else is in a better position to guide research then the young service users themselves? It is not the young people’s (both professionals’ and service users’) fault that global mental health remains a neglected area of clinical research and service development. Yet they will have to transform it. Let’s give them the chance.
